# Allogeneic cell therapy using umbilical cord MSCs on collagen scaffolds for patients with recurrent uterine adhesion: a phase I clinical trial

**DOI:** 10.1186/s13287-018-0904-3

**Published:** 2018-07-11

**Authors:** Yun Cao, Haixiang Sun, Hui Zhu, Xianghong Zhu, Xiaoqiu Tang, Guijun Yan, Jingmei Wang, Donghui Bai, Juan Wang, Liu Wang, Qi Zhou, Huiyan Wang, Chengyan Dai, Lijun Ding, Biyun Xu, Yan Zhou, Jie Hao, Jianwu Dai, Yali Hu

**Affiliations:** 1 The Affiliated Drum Tower Hospital of Nanjing University Medical School, Nanjing, China; 2 Institute of Zoology, Chinese Academy of Sciences, 1 West Beichen Road, Chaoyang district, Beijing, 100101 China; 3 Institute of Genetics and Developmental Biology, Chinese Academy of Sciences, 3 Nanyitiao, Zhongguancun, Beijing, 100190 China; 4 University of California, San Francisco, CA USA; 5 Department of Obstetrics and Gynecology, Nanjing Drum Tower Hospital and Jiangsu Key Laboratory for Molecular Medicine, Nanjing University Medical School, 321 Zhongshan Road, Nanjing, China

**Keywords:** Asherman syndrome, Intrauterine adhesions, Uterine infertility, UC-MSCs, Collagen scaffold

## Abstract

**Background:**

Intrauterine adhesions (IUA) are the most common cause of uterine infertility and are caused by endometrium fibrotic regeneration following severe damage to the endometrium. Although current stem cell treatment options using different types of autologous stem cells have exhibited some beneficial outcomes in IUA patients, the reported drawbacks include variable therapeutic efficacies, invasiveness and treatment unavailability. Therefore, the development of new therapeutic stem cell treatments is critical to improving clinical outcomes.

**Methods:**

Twenty-six patients who suffered from infertility caused by recurrent IUA were enrolled in this prospective, non-controlled, phase I clinical trial with a 30-month follow-up. During the procedure, 1 × 10^7^ umbilical cord-derived mesenchymal stromal cells (UC-MSCs), loaded onto a collagen scaffold, were transplanted into the uterine cavity following an adhesion separation procedure. Medical history, physical examination, endometrial thickness, intrauterine adhesion score and the biological molecules related to endometrial proliferation and differentiation were assessed both before and 3 months after cell therapy.

**Results:**

No treatment-related serious adverse events were found. Three months after the operation, the average maximum endometrial thickness in patients increased, and the intrauterine adhesion score decreased compared to those before the treatment. A histological study showed the upregulation of ERα (estrogen receptor α), vimentin, Ki67 and vWF (von Willebrand factor) expression levels and the downregulation of ΔNP63 expression level, which indicates an improvement in endometrial proliferation, differentiation and neovascularization following treatment. DNA short tandem repeat (STR) analysis showed that the regenerated endometrium contained patient DNA only. By the end of the 30-month follow-up period, ten of the 26 patients had become pregnant, and eight of them had delivered live babies with no obvious birth defects and without placental complications, one patient in the third trimester of pregnancy, and one had a spontaneous abortion at 7 weeks.

**Conclusions:**

Transplanting clinical-grade UC-MSCs loaded onto a degradable collagen scaffold into the uterine cavity of patients with recurrent IUA following adhesiolysis surgery is a safety and effective therapeutic method.

**Trial registration:**

Clinicaltrials.gov. NCT02313415, Registered December 6, 2014.

**Electronic supplementary material:**

The online version of this article (10.1186/s13287-018-0904-3) contains supplementary material, which is available to authorized users.

## Background

Asherman syndrome (AS) is characterized by severe damage of the endometrium due to curettage and/or endometritis [1]. Approximately 25–30% of infertile women suffer from AS, which represents the most common cause of uterine infertility [2]. The traditional treatment for AS is the transcervical resection of adhesion by hysteroscopy followed by the placement of an intrauterine device, Foley catheter or biomaterials to prevent recurrent intrauterine adhesions (IUA) [3, 4] and hormonal therapy to regenerate the endometrium [5]. However, in severe cases, the incidence of recurrent IUA was reported to be as high as 62.5% [6]. This endometrial fibrosis impedes embryo implantation [7]. Therefore, preventing IUA recurrence and endometrial scarring are considered key factors that affect therapeutic outcomes [8].

Stem cells have been shown to contribute to the repair and regeneration of the endometrium [9]. Some small case studies and case reports have documented that the injection of autologous stem cells (from bone marrow, peripheral blood or menstrual blood) into the uterine wall or into uterine blood vessels can improve endometrium regeneration in AS patients [10–12]. Of note, the quantity and quality of autologous stem cells have varied among patients and with different processing methods, which can influence the therapeutic outcomes of patients. Because of the safety and efficacy of using umbilical cord-derived mesenchymal stromal cells (UC-MSCs) to support the regeneration of damaged tissues [13, 14], we developed a novel therapy using clinical-grade UC-MSCs to treat AS patients. In this treatment, a complex of UC-MSCs supported by a degradable collagen scaffold was transplanted into the uterine cavity of post-adhesiolysis surgical patients. By gathering stem cells, maintaining their viability [15], and increasing the duration of contact with the damaged site of the endometrium [16], the capacity for endometrial proliferation and differentiation was improved significantly. Here, we report the results of this phase I clinical trial.

## Methods

### Study enrollment and inclusion and exclusion criteria

These findings are the mid-term results of a prospective, experimental, non-controlled, phase I clinical trial supported by investigator-driven funding. The inclusion criteria included secondary infertility or embryo transfer failure caused by recurrent IUA, a desire to be pregnant, an age of less than 45 years, and agreeing to participate in the study. Patients were excluded from recruitment if they had any of the following issues: having hysteroscopy contraindications, chromosome karyotype abnormalities, congenital uterine malformations, severe adenomyosis, contraindications to pregnancy, contraindications to estradiol treatment, medical history of pelvic malignant tumors, previous experience with pelvic radiotherapy. For patients who met the indications for stem cell therapy, written informed consent was obtained. Patients with recurrent IUA were diagnosed according to their history profiles, and three experienced gynecologists at the Drum Tower Hospital performed hysteroscopy examinations for severity classification using the American Fertility Society scoring method (The American Fertility Society, 1988) during the screening process.

### Preparation of the UC-MSC /collagen complex

The regeneration-induced functional complex was made as follows: a 4 cm × 6 cm collagen scaffold with pores of 20–200 μm in diameter [17] (Zhenghai Biotechnology Company, Shandong, China) was rinsed with xeno-free MSC culture medium (MesenCult™ MSC Basal Medium, Stemcell Technologies, Vancouver, Canada), excess fluid was aspirated, and a suspension of 1 × 10^7^ (about 4.2 × 10^5^/cm^2^) UC-MSCs was dripped uniformly onto the scaffold. The cell-seeded scaffold was incubated in humid air consisting of 5% CO_2_ at 37 °C for 1 h before transplantation.

### Hysteroscopic operations

Two experienced gynecologists using ultrasound guidance performed the hysteroscopic operations. The endometrial adhesions were separated using non-electrified micro scissors until an anatomical uterine cavity with slight staxis was observed. The UC-MSC/collagen scaffold complex was spread onto an 18F Foley catheter and placed into the uterine cavity, and then an infill catheter bulb containing 3 ml of saline was used to attach the scaffold to the inner wall of uterine cavity. After 12 h, the catheter was removed after withdrawing saline in the bulb. The procedure was performed following 10 days of 6 mg/day Progynova (estradiol) (menstrual period day 13). Continuous administration of the same dosage of Progynova, lasting for 30 days following the operation, and 60 mg of progesterone was injected on the 30th day post-operation. Then, the hormone replacement therapy was stopped, and patients returned to a natural menstrual cycle.

### Follow-up

#### Adverse events and safety assessment

Antibiotics were used to prevent infection in all patients 30 min before surgery and 3 days after surgery. Surgical complications (uterine perforation, anesthesia accidents), body temperature, hemogram (leukocyte count, serum C-reactive protein, erythrocyte sedimentation rate), and vaginal discharge after the operation were recorded.

#### Patient-reported outcomes

The volume of menstruation was recorded by all patients according to a pictorial chart [18] before and after surgery. An assessment of menstruation recovery was made using these data.

#### Transvaginal sonography

Baseline (during the natural menstrual cycle before surgery) and follow-up (3 months after surgery) endometrial thickness and blood flow measurements were taken using a Doppler ultrasound scan to evaluate endometrial regeneration. We also assessed the degradation of the collagen scaffold in the second month following surgery. The image pulse repetition frequency (PRF) was 0.6 kHz when assessing blood flow.

#### Endometrial biopsy and second-look hysteroscopy

Hysteroscopic inspections were performed at the late proliferation phase, endometrial biopsies were collected from the adhesion area, and the locations were recorded prior to treatment with UC-MSCs. A second-look hysteroscopic inspection was performed still at the late proliferation phase, 3 months after UC-MSC transplantation, by the same gynecologist. The IUA score and the anatomy of the uterine cavity were evaluated during this hysteroscopic inspection. At the same time, endometrial biopsies were performed by sampling endometrial tissues at the location of the first biopsies.

#### Pregnancy outcome

If the anatomy of the uterine cavity was normal 3 months after the operation, embryo transfer or spontaneous pregnancy was prepared for these patients. The pregnant patients were followed until the end of pregnancy, with fetal structure screening, aneuploidy screening and routine prenatal visits. Placental complications were monitored by ultrasound examinations during the pregnant period and by pathologic examinations following placenta delivery. All patients were followed until the end of August 2017.

#### Efficacy assessment: primary outcome measures

The primary outcomes were the IUA score and the maximum endometrial thickness. Secondary outcomes included live birth rate, ongoing pregnancy rate (> 12 weeks active fetus), miscarriage rate, menstrual volume and histological changes in the endometrium. The IUA score and endometrial thickness were assessed both before and 3 months after treatment. The endometrial biopsies obtained before and after treatment were stained for ΔNp63, Ki67, estrogen receptor alpha (ERα), and von Willebrand factor (vWF), and quantification of positive staining was performed to evaluate endometrial molecular features.

#### Hematoxylin-eosin staining and immunohistochemical analysis

The sections (5 μm) on slides were immersed in xylene (3 min, twice), and rehydrated in a decreasing ethanol series diluted in distilled water (100%, 100%, 95%, 95%, 75%, 0%, 1 min each). The sections were rinsed in water, stained in hematoxylin for 45 s, and rinsed in water. After the staining, sections were air-dried in room and mounted using Permount™ Mounting Medium.

Immunohistochemical staining was used to assess the regeneration and neovascularization of the endometrium. Paraffin blocks were cut at a thickness of 2 μm. Serial sections were collected on polylysine-coated glass slides and deparaffinized, and endogenous peroxidase activity was blocked with 3% H_2_O_2_. After heat-mediated antigen retrieval, the slides were incubated with 10% serum from the species in which the secondary antibody was generated to block nonspecific binding. The tissue sections were labeled with antibodies (ER-α, Epitomic EP1; Ki67, Abcam ab15580, Cambridge, UK; vWF, Abcam ab6994, Cambridge, UK; ΔNp63, Zytomed MSK097–05, Berlin, Germany) overnight at 4 °C. Negative controls were performed by substituting the primary antibodies with the same concentration of pre-immune rabbit or mouse IgG or by omitting the primary antibodies. After incubation with HRP-conjugated secondary antibodies, the sections were exposed to DAB to visualize the antigen signals. The sections were counterstained with hematoxylin and viewed under a microscope (DMi8, Leica, Wetzlar, Germany). Positively stained cells were counted or stain density was measured in all fields for each slide.

#### Short tandem repeat (STR) analysis

Genomic DNA was extracted from the UC-MSCs and endometrial biopsies 3 months after surgery from ten patients. The loci D1S2344, D1S104, D1S2740, D5S681, D5S1977, D7S820, D13S317, D2S1338, and D21S111 were amplified and electrophoresed by genetic analyzer 3730 (Applied Biosystems, Thermo Fisher Scientific, Waltham, MA, USA). The lengths of the amplicons were compared between the donor and the repaired tissues. The percentage of the donor DNA was calculated based on the areas of the alleles. To provide positive controls, we made two mixed DNAs with two unrelated DNAs, which were selected from our laboratory with highly recognizable STR alleles, with 1:9 and 1:5 as a recipe.

#### Statistical analysis

The analysis was performed based on the intention-to-treat (ITT) and per protocol principles. Statistical comparisons were performed using a paired-samples *t* test, and the rank-sum test was performed using the Statistical Program for Social Sciences (IBM SPSS, Inc., Version 21.0, IBM Corp., Armonk, NY, USA). A two-sided *P* < 0.05 was considered to be statistically significant.

## Results

### Participant flow

**Fig 1:**
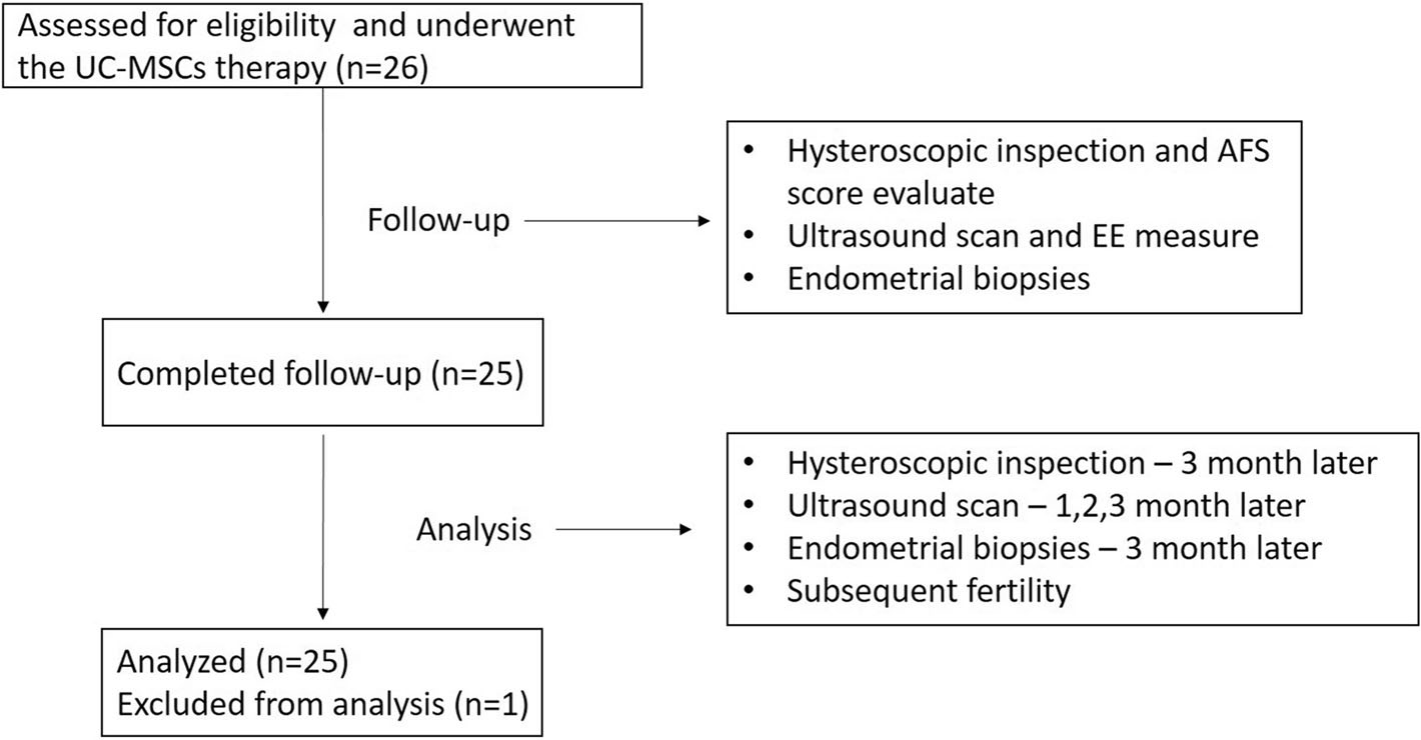
A flow chart of the patient enrollment process. *UC-MSC* umbilical cord-derived mesenchymal stem cells

Twenty-six patients with recurrent IUA were assessed for the study over 3 months following the steps outlined in the flow chart (Fig. 1). Ultimately, 25 patients remained in the study cohort for the duration of the 30-month follow-up period.

### Clinical-grade human UC-MSCs

The clinical-grade UC-MSCs were isolated and cultured in a GMP workshop (Additional file 1: Supplementary material). A flow chart demonstrating the bioprocesses utilized during UC-MSC preparation for clinical use was designed and included cell isolation, expansion, characterization, certification, and safety assessment prior to transplantation (Additional file 2: Figure S1A). Homogeneous, fibroblastic-shaped cells were observed under a microscope during the process of cell expansion (Additional file 2: Figure S1B). Phenotypic analysis and differentiation experiments were performed from P9 to P14 to characterize the UC-MSCs (Additional file 2: Figure S1A, C and D). Flow cytometry analysis showed that the purity of the UC-MSCs was greater than 98%, which was confirmed by the high positive rates of CD105, CD73, CD90 and CD29, while the hematopoietic markers CD45 and CD34 were negative (Additional file 2: Figure S1C). Immunofluorescence staining results demonstrated the specific expression of FABP, osteocalcin and aggrecan in isolated cells (Additional file 2: Figure S1D). Immunohistochemical staining using Oil-red O, Alizarin Red and Alcian blue exhibited the presence of lipids, mineralized plaques and cartilaginous substrates derived from hUC-MSCs, respectively (Additional file 2: Figure S1D). UC-MSCs (P10/P15/P20) were subjected to the National Institutes for Food and Drug Control (NIFDC) guidelines for a series of biosafety evaluations and those results are summarized in Additional file 3: Table S1. The cells were accredited by the NIFDC (report number SH201401380), according to Chinese regulations.

### Baseline characteristics of the patients

All 26 patients had experienced at least one unsuccessful hysteroscopic adhesiolysis attempt, and the average of 2.34 attempted hysteroscopy surgeries. Specifically, ten patients experienced one hysteroscopic attempt, nine patients experienced two hysteroscopic attempts, four patients experienced four hysteroscopic attempts, one patient experienced five hysteroscopic attempts, and two patients experienced more than five hysteroscopic attempts.

The clinical characteristics of the patients enrolled in the study are shown in Table 1. The average age of the patients was 35.1 ± 3.8 years old (patient ages ranged from 27 to 42 years old) at the time of intervention. Eleven patients were over 35 years old, and 15 patients were under 35 years old. Patients had an average duration of infertility of 4.46 **±** 2.73 years (ranging from 2 to 16 years). The primary etiology of IUA was dilatation and curettage. Twenty-three patients presented hypomenorrhea, one patient had amenorrhea, and two patients had normal menstrual histories. All 26 patients had been diagnosed as severe AS during previous surgeries. In the current hysterscopic inspections, seven patients were evaluated as moderate AS (6–8 scores), and the remaining 19 patients were categorized as severe AS based on the AFS classification system (10 scores).

**Table 1:** Clinical characteristics and outcome of patients

Patient	Age (years)	Symptoms	Etiology	Prior repair attempts	Hysteroscopy (IUA score) pre-/3-month post-UC-MSC therapy	Pregnancy outcome
P1	34	Infertility (5 years), Hypomenorrhea	3 D&C	2 HSP	10/4	Spontaneous pregnancy 21 months post treatment and cesarean section at 37^+ 6^ weeks, boy, 3300 g
P2	34	Infertility (5 years), Hypomenorrhea	4 D&C	2 HSP	10/8	
P3	39	Infertility (7 years), Hypomenorrhea	7 D&C	1 HSP	6/0	IVF-ET and implantation failure
P4	33	Infertility (2 years), Hypomenorrhea	6 D&C	2 HSP	10/6	Spontaneous pregnancy 16 months post treatment and cesarean section at 38^+ 1^ weeks, girl, 3200 g
P5	33	Infertility (2 years), Hypomenorrhea	1 D&C	1 HSP	6/4	Spontaneous pregnancy 11 months post treatment and cesarean section at 29^+ 5^ weeks, boy, 1690 g
P6	33	Infertility (14 years), Hypomenorrhea	Unknown	2 HSP	10/10	
P7	42	Infertility (2 years), Hypomenorrhea	1 D&C	2 HSP	10/7	
P8	37	Infertility (2 years), Hypomenorrhea	7 D&C	4 HSP	10/4	
P9	27	Infertility (5 years), Hypomenorrhea	3 D&C	5 HSP	10/10	
P10	31	Infertility (2 years), Hypomenorrhea	spontaneous abortion	2 HSP	10/5	IVF-ET 16 months post treatment and cesarean section at 37^+ 5^ weeks, girl, 3400 g
P11	34	Infertility (8 years), Hypomenorrhea	Unknown	4 HSP	10/10	
P12	30	Infertility (4 years), Hypomenorrhea	2 D&C	2 HSP	10/8	
P13	41	Infertility (2 years), Hypomenorrhea	2 D&C	6 HSP	8/4	
P14	40	Infertility (8 years), Hypomenorrhea	3 D&C	1 HSP	10/6	
P15	34	Infertility (2 years), Amenorrhea	4 D&C	1 HSP	10/0	Spontaneous pregnancy 20 months post treatment and delivered at 40^+ 4^ weeks, boy, 3450 g
P16	34	Infertility (3 years), Hypomenorrhea	spontaneous abortion	1 HSP	10/7	IVF-ET and implantation failure
P17	33	Infertility (5 years), Hypomenorrhea	4 D&C	6 HSP	7/3	IVF-ET 5 months post treatment and cesarean section at 37^+ 5^ weeks, boy, 3600 g
P18	32	Infertility (6 years), Hypomenorrhea	3 D&C	1 HSP	7/0	IVF-ET 15 months post treatment and cesarean section at 27^+ 3^ weeks, boy, 1100 g
P19	37	Infertility (5 years), Hypomenorrhea	10 D&C	2 HSP	10/8	Spontaneous pregnancy 24 months post treatment and ongoing third trimester pregnancy
P20	40	Infertility (3 years), Hypomenorrhea	2 D&C	1 HSP	8/0	Spontaneous pregnancy 10 months post treatment and cesarean section at 37^+ 4^ weeks, girl, 3930 g
P21	33	Infertility (2 years), Hypomenorrhea	4 D&C	1 HSP	10/4	
P22	40	Infertility (3 years), Normal menstruation	7 D&C	2 HSP	10/8	
P23	35	Infertility (5 years), Hypomenorrhea	5 D&C	1 HSP	10/10	Spontaneous pregnancy 26 months post treatment and abortion at 7th week
P24	30	Infertility (3 years), Hypomenorrhea	3 D&C	4 HSP	10/6	
P25	36	Infertility (6 years), Normal menstruation	2 D&C	1 HSP	6/6	
P26	34	Infertility (5 years), Hypomenorrhea	1 D&C	4 HSP	10/refused to follow-up	

*D&C* dilatation and curettage, *HSP* hysteroscopic adhesiolysis, *IVF-ET* in vitro fertilization-embryo transfer

All patients immediately reported longer and heavier menstruation in the first menstrual cycle following UC-MSC transplantation, and the patient with amenorrhea (Patient No. 15) recovered menstruation following cell therapy.

### Adverse events and safety assessment

In order to assess the safety of the treatment, we determined the surgical complications, systemic and local safety after the surgery. No surgical complications were identified during the surgery. Body temperatures, hemograms, and vaginal discharge following the operation were normal. The average of white blood cell count (5.96 **±** 1.46 10^9^/L), neutrophil percentage (52.2 **±** 8.99%) and C-reactive protein (2.27 **±** 0.43 mg/L) at 1 day after surgery were presented to be normal (Additional file 4: Table S2). No inflammation reaction was detected from the endometrial biopsies 3 months after surgery (Additional file 5: Figure S4). Therefore, no adverse events occurred throughout the observation period in all patients.

### Second-look hysteroscopic inspections

**Fig. 2:**
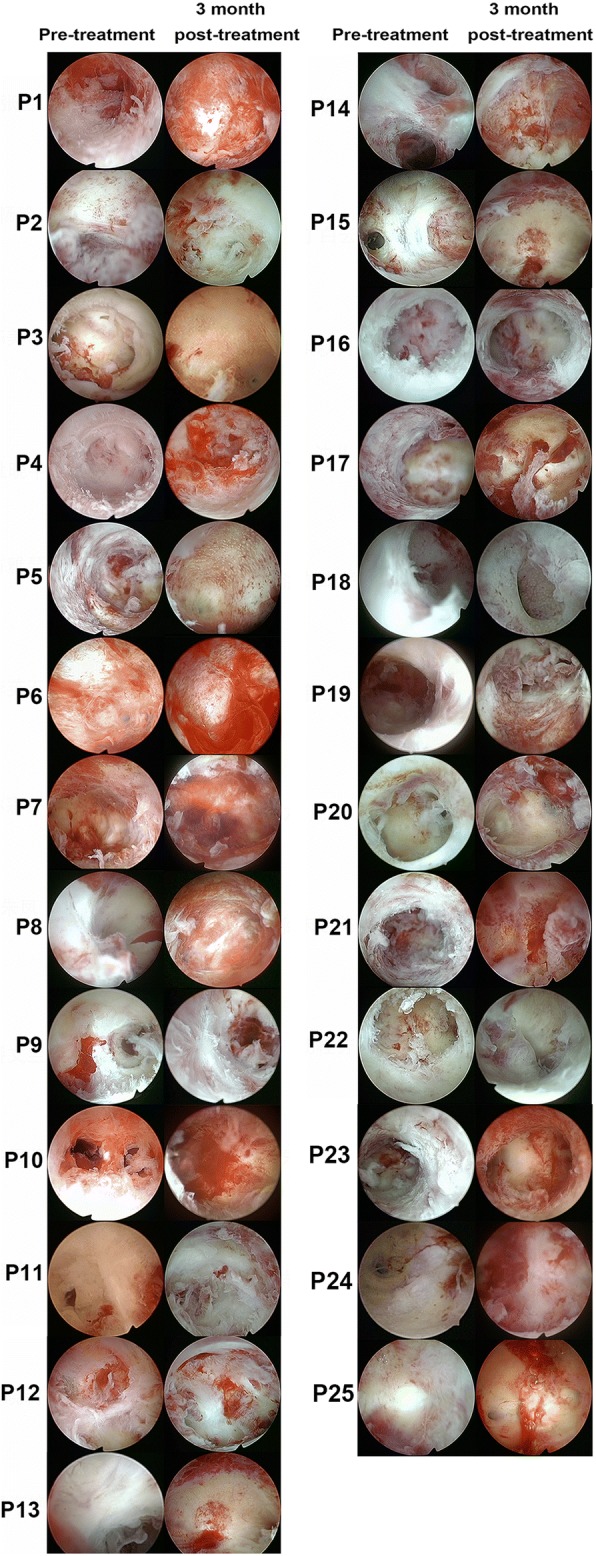
Hysteroscopic inspection images from all 25 patients, before and after UC-MSC/collagen treatment

The second-look hysteroscopic inspections were performed in 25 patients at 3 months after the procedure (Fig. 2). Comparing the inspections performed before and after the surgery, the mean IUA score of the 25 patients decreased from 9.12 ± 1.51 to 5.52 ± 3.22. Particularly, ten patients were found to have normal uterine cavities, of whom four patients had no intrauterine adhesion following cell therapy (Patient Nos. 3, 15, 18, 20), and six patients showed mild adhesion. Ten patients recovered from severe AS to moderate AS, and the remaining five patients showed no improvement (Fig. 2).

### Ultrasound scans

**Fig. 3:**
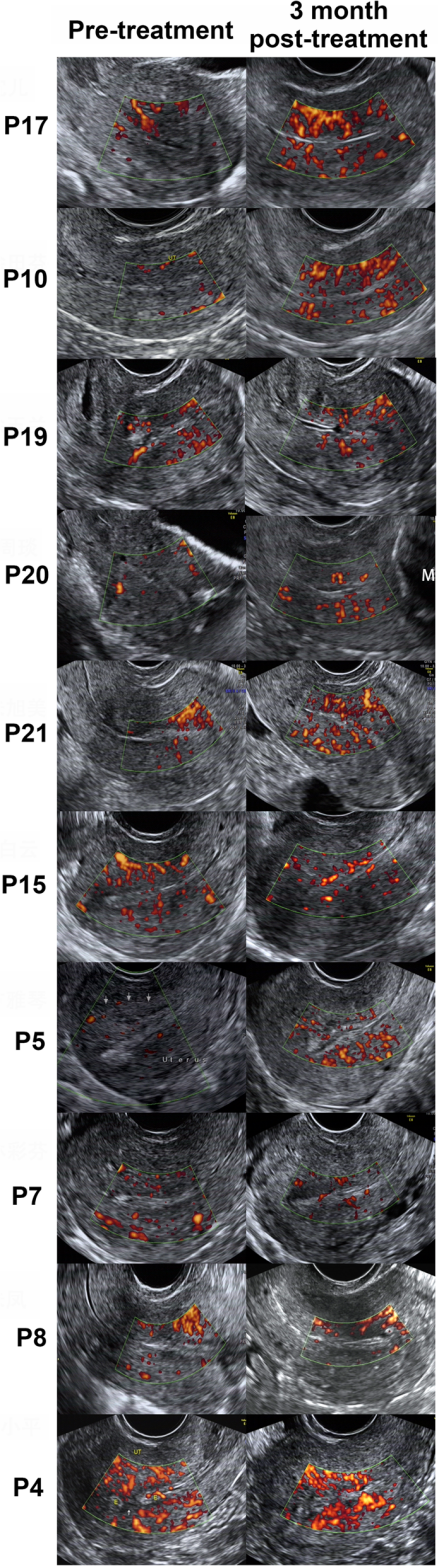
Blood flow of ten patients before and after UC-MSC/collagen treatment by Doppler ultrasound

The ultrasound results revealed the degradation of the collagen scaffold at the end of the second month following surgery for all patients, and significant improvements in the endometrial thickness and blood flow were found at the end of the third month (Fig. 3). The average maximum endometrial thickness, measured in 25 patients, increased from 4.46 ± 0.85 mm to 5.74 ± 1.20 mm (*P* < 0.01, Additional file 6: Figure S2).

### Histological studies

**Fig. 4:**
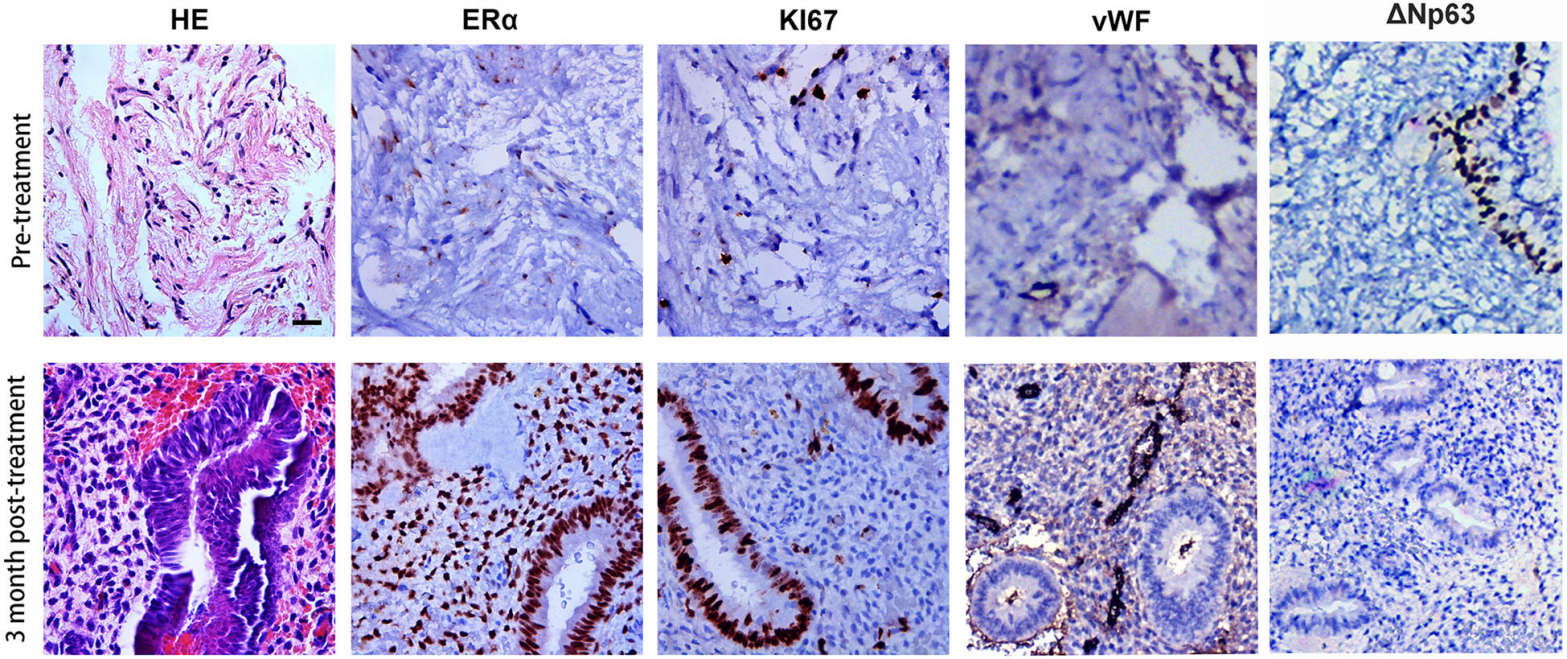
Immunohistochemical staining of ERα, Ki67, vWF and ΔNp63 on endometrial biopsy samples obtained from patients before and after UC-MSC/collagen treatment. Scale bar: 100 μm. *ERα* estrogen receptor alpha, *vWF* von Willebrand factor

In this study, endometrial biopsies from the first and second hysteroscopic inspections in 25 patients were studied to determine the histological and molecular changes before and after the treatment. We detected the ectopic expression of ΔNp63 in the endometria and showed 16 of 25 patients had ΔNp63-positive endometrial epithelial cells prior to treatment, which was consistent with our previous report [16]. After treatment, 11 patients showed decreased or undetectable levels of ΔNp63 expression. Upregulated ERα, Ki67 and vWF expression levels (Fig. 4) after treatment indicated the improvement of differentiation, proliferation and neoangiogenesis of endometria (Additional file 7: Figure S3 A-D).

### Short tandem repeat analysis

At least five (5–8) loci could be used to define the origin of the genomic DNA from the biopsies. For the two positive reference DNA mix samples, the “donor” peak could be easily recognized in proportion. No allele peaks of the allogeneic MSCs appeared in the endometria taken 3 months after treatment according to the electrophograms.

### UC-MSC/scaffold transplantation improved the pregnancy rate in patients with recurrent IUA

If no obvious adhesion was found during the second hysteroscopic inspection, an embryo transfer (ET) or spontaneous pregnancy was planned for these patients. Among them, three patients became pregnant after ET (Patient Nos. 10, 17, and 18), seven patients became pregnant spontaneously (Patient Nos. 1, 4, 5, 15, 19, 20, and 23), and two patients experienced failed ET (Patient Nos. 3 and 16) (Table 1).

In total, ten patients became pregnant by the end of 30-month follow-up period. By the end of August 2017, eight patients had given live birth, one patient was still in the third trimester of pregnancy (Patient No. 19), and one patient suffered from spontaneous abortion in the seventh week (Patient No. 23). Patients gave birth by cesarean section at term, except for Patient Nos. 5 and 18 who both experienced preterm birth due to premature rupture of the membrane. No placental complications were found in the patients who have delivered. All babies (five boys and three girls) were healthy, including the two preterm babies.

## Discussion

Hysteroscopic adhesiolysis is a common treatment option for patients with IUA. The successful rate of hysteroscopic treatment varies in previous literature. High grades of adhesions are predictive of an increased risk for the spontaneous recurrence of adhesions [1, 8]. For patients with severe recurrent AS, hysteroscopic treatment failure may represent refractory uterine infertility.

Alternative salvage methods have been investigated. Stem cells targeting endometrial regeneration have been reported and investigated since 2007 [19].

In 2011, a case study reporting the placement of autologous BMSCs into the uterine cavity after curettage showed that the transplantation increased endometrial proliferation in a severe AS patient, who was then able to experience 8 weeks of pregnancy [20]. In 2014, a report on the subendometrial injection of CD34+ or cultured autologous BMCs in severe AS patients showed a slight increase in the endometrial thickness [12]. In 2016, a case study was reported on 16 patients with refractory infertility who received autologous peripheral blood CD133+ cells into the spiral arteries of the uterus and were observed to experience endometrial reconstruction; two of the 16 patients with AS and one patient with a thin endometrium had live births [11]. In the same year, a report on the transplantation of autologous menstrual blood-derived stromal cells into seven severe AS patients showed that two of them successfully conceived. In our previous study, we reported a successful transplanting of autologous mononuclear cells from the bone marrow/collagen complex into patients with severe IUA, who achieved pregnancies and live births after treatment [16]. In the current study, we report the results of the first phase I clinical trial of a clinical use-approved UC-MSC/collagen scaffold for the treatment of recurrent IUA. We demonstrate the clinical safety and efficacy of a clinical-grade UC-MSC/collagen scaffold. The UC-MSCs were obtained noninvasively. The hypoimmunogenic and nontumorigenic features of UC-MSCs make them especially advantageous for use in regenerative treatment [19, 21–24]. Therefore, UC-MSCs can occupy an intermediate position between the most versatile pluripotent ESCs/iPSCs and adult tissue-specific MSCs [25]. Finally, but more importantly, it is easy to ensure quality control during the standardized production of UC-MSCs.

The uterus is a hollow viscera, which makes it difficult to provide an attachment site for stem cells during cell therapy. Most of the previous stem cell treatments employed the methods of intravenous injection [26] or uterine artery injection [11]. Under those conditions, it is difficult for any type of stem cell to colonize within the uterus, weakening the regenerative effects of stem cells. Although a few studies have reported that stem cells can be injected into the basalis layer of the endometrium when guided by ultrasound [12], it is difficult to maintain those cells in the correct layer and to cover the entire inner surface of the uterus. In our study, we took advantage of a collagen scaffold to provide an attachment site for the MSCs and to maintain a high density of UC-MSCs at the injury site to improve endometrial regeneration. According to our STR results, the role of UC-MSC/collagen in endometrial regeneration may be related to the improvement of multiple factors within the regenerative environment instead of directly participating in the reconstruction of the endometrium. This result was in accordance with finding reported by de Windt et al. [21]。 Ectopic ΔNp63 expression in endometrial epithelial cells inhibits the proliferation and differentiation of the endometrium in IUA [16]. In the present study, UC-MSC/collagen downregulated ΔNp63 expression levels, which may be a contributing factor for the regeneration of the endometrium.

## Conclusions

This study showed the safety and efficacy of transplanting clinical-grade UC-MSCs loaded onto a degradable collagen scaffold into the uterine cavity following adhesiolysis surgery for recurrent IUA patients. By gathering stem cells, maintaining their viability, and increasing the duration of contact with the damaged site of the endometrium, the capacity for endometrial proliferation and differentiation was improved significantly by the use of a scaffold. These results indicate a promising future for patients with AS for whom hysteroscopic treatment has failed.

## Additional files

Additional file 1: Supplementary material. (DOCX 17 kb)

Additional file 2: **Figure S1. (**A) A flow chart of UC-MSC preparation for clinical use, including cell isolation, expansion, characterization, certification and safety assessment prior to transplantation. (B) Cell shapes at passages 1, 6 and 15. (C) Flow cytometry analysis of UC-MSCs showed high positive rates of CD105, CD73, CD90 and CD29, while CD45 and CD34 were negative. (D) Immunofluorescence of FABP, osteocalcin and aggrecan and immunohistochemical staining using Oil-red O, Alizarin Red and Alcian blue exhibited the presence of lipids, mineralized plaques and cartilaginous substrates derived from UC-MSCs, respectively. (TIF 4967 kb)

Additional file 3: **Table S1.** Biological safety and biological activity analysis of the clinical-grade cells^**$**^ recognized by the National Institutes for Food and Drug Control (NIFDC). $: This table is translated from NIFDC report numbers, SH20140138 for UC-MSC. (DOCX 21 kb)

Additional file 4: **Table S2.** Serum levels of C-reactive protein, leukocyte count and neutrophil percentage at 1 day after surgery in each patient. (DOCX 14 kb)

Additional file 5: **Figure S4.** H&E staining of endometrium biopsies from all 25 patients after UC-MSC/collagen treatment (TIF 15601 kb)

Additional file 6: **Figure S2.** The average maximum endometrial thickness in patients before and after UC-MSC/collagen treatment measured by ultrasound. *n* = 25, **P < 0.01 (TIF 3871 kb)

Additional file 7: **Figure S3. (**A-B) The positive staining ratio between ERα and Ki67 in endometrial biopsies from patients before and after UC-MSC/collagen treatment. (C) The average number of microvessels in each image of endometrial biopsies from patients before and after UC-MSC/collagen treatment. (D) The number of ΔNp63-positive cells counted in endometrial biopsies from patients before and after UC-MSC/collagen treatment. n = 25 samples, verified by two technicians, *P < 0.05, ***P < 0.001 (TIF 13448 kb)

## Abbreviations

AS: Asherman syndrome
ERα: Estrogen receptor alpha
ET: Embryo transfer
IUA: Intrauterine adhesions
NIFDC: National Institutes for Food and Drug Control
UC-MSCs: Umbilical cord-derived mesenchymal stem cells
vWF: von Willebrand factor
