# Supplementary material for: Allogeneic cell therapy using umbilical cord MSCs on collagen scaffolds for patients with recurrent uterine adhesion: a phase I clinical trial
Source: Stem Cell Res Ther. 2018 Jul 11;9:192. doi: 10.1186/s13287-018-0904-3 (PMC6042450; doi:10.1186/s13287-018-0904-3)
Supplement: Supplementary file 1 — Supplementary material. (DOCX 17 kb) [file 13287_2018_904_MOESM1_ESM.docx]

1. Isolation of UC-MSCs

Human newborn umbilical cord tissues were conserved into sterile phosphate-buffered saline (PBS，Gibco,14190-136 ) containing 2% of penicillin / streptomycin (SP，Gibco,15140-122 ) and transported on ice. UC tissues were sectioned into 2-cm sections and washed twice in PBS to remove contaminating blood cells. Umbilical arteries and vein were removed, and the remaining tissues were cut into 1-mm3 pieces. Tissue fragments were attached to T225 cell culture flasks, cultured in xeno-free MSC culture medium (MesenCult™ MSC Basal Medium + MesenCult™ MSC Stimulatory Supplement, STEMCELL Technologies) and then placed into 37℃, 5% CO2 incubator. Medium was changed every two days. Adherent cells could be observed and were passaged after 7 or 10 days.

1. Passage of UC-MSCs

Medium was discarded and Cells were washed with PBS at room temperature. Pre-warmed Tryple was used to digest UC-MSCs for 2 to 3 minutes at 37 ℃. Digestion was terminated by MSC culture medium. Cells were resuspended by fresh MSC culture medium, counted and then seeded into 100-mm cell culture dishes at 1 × 10^6^ cells/dish. Cultures were placed into 37℃, 5% CO2 incubator.

1. Biosafety evaluation

The tested items are listed in Table S1. The “Pharmacopoeia of the People's Republic of China, Edition 2010, VolumeIII” was used as a reference for the testing methods.

1. Cryopreservation of UC-MSCs

UC-MSCs were digested as described in Passage of UC-MSCs. Serum-free and animal component- free cryomedia, STEM-CELLBANKER GMP (CELLBANKER) was added to resuspend and subpackage cells into frozen vials. After freezing in Nalge Nunc Cryo containers overnight in a freezer at 80 ℃, the vials were transported in the gas phase of a liquid nitrogen tank.

1. UC-MSC Thawing

Cell suspension stored in frozen tubes was thawed in 37℃ water bath, and then quickly transferred into 15-ml centrifuge tubes containing pre-warmed MSC culture medium. Cells were resuspended by fresh MSC culture medium after centrifugation, counted and then seeded into 100-mm cell culture dishes at 1 × 106 cells/dish. Cultures were placed into 37℃, 5% CO2 incubator.

1. Adipogenic, osteogenic and chondrogenic differentiation

For adipogenic differentiation, UC-MSCs were digested by 0.05% trypsin as described before, resuspended by fresh MSC culture medium and seeded into 4-well plates containing slides at 4 × 104 cells/well. MSC culture medium was replaced by adipogenic differentiation medium （R&D, SC006，prepared as instruction manual told） at 100% confluence. Fat vesicles could be observed 1 to 3 weeks later. For osteogenic differentiation, UC-MSCs were digested by 0.05% trypsin as described before, resuspended by fresh MSC culture medium and seeded into 4-well plates containing slides at 1 × 104 cells/well. MSC culture medium was replaced by osteogenic differentiation medium （R&D, SC006，prepared as instruction manual told） at 70% confluence. Calcium deposition could be observed 3 weeks later. For chondrogenic differentiation, UC-MSCs were digested by 0.05% trypsin as described before, resuspended by fresh MSC culture medium and seeded into 15-ml centrifuge tubes at 2.5 × 105 cells/tube. MSC culture medium was replaced by chondrogenic differentiation medium (without supplement), and then 500 μl of chondrogenic differentiation medium （R&D, SC006，prepared as instruction manual told） was used to resuspend UC-MSCs. 15-ml centrifuge tubes containing cells were placed into incubators after centrifugation (without discarding supernatant), and cultured for 3 weeks. Immunohistochemical staining was carried out 21 days after differentiation, including Oil-red O (ALORICH, 234117), Alizarin Red (Sigma, A3882) and Alcian Blue (Sigma, A5268).

1. Immunofluorescence staining

Immunofluorescence staining was performed as previously described^26^. Cell samples were fixed with 4% (w/v) paraformaldehyde (PFA, P6147, Sigma) for 10 minutes and then permeabilized with 0.5% Triton X-100 (t8200, Solarbio) and blocked in 2% bovine serum albumin (BSA, A7906, Sigma) at 37°C for 10 minutes after rinsing. Samples were subsequently incubated with primary antibodies against FABP (Pierce, PA5-30591, 1:200), Osteocalcin (Pierce, PA5-11849, 1:200), Aggrecan (Pierce, MA3-16888, 1:200), at 4°C overnight. On day 2, the samples were rinsed and then incubated with the appropriate secondary antibodies donkey-anti-mouse-cy5 (Jackson ImmunoResearch, 715-605-151, 1:200), donkey-anti-goat-Cy5 (Jackson ImmunoResearch, 705-605-147, 1:200), or donkey-mouse-cy3 (Jackson ImmunoResearch, 715-165-150, 1:200) for 1 hour at 37°C. Nuclei were visualized by staining with Hoechst 33342 (10 μg/mL) or propidium iodide (PI, 5 μg/mL) for 10 minutes at room temperature.

1. Flow cytometry

Cells were dissociated into single cells and then fixed with 4% PFA for 15 minutes at room temperature. After permeabilization in 0.1% Triton X-100 for 30 minutes at room temperature, the cells were stained with primary antibodies, followed by secondary antibodies diluted in PBS plus 2% BSA. Data were collected on the flow cytometer and analyzed using FlowJo software. Anti-human CD29 (BioLegend, 303004), anti-human CD105 (BioLegend, 323206), anti-human CD73 (BD, 550257), anti-human CD34 (BD, 555822), anti-human CD45 (eBioscience, 11-9459-42) were used. UC-MSCs only treated with secondary antibodies were used as a gating control in the experiments of pluripotency characterization. Undifferentiated UC-MSCs treated with the same antibodies were used as negative control for gating in differentiation experiments.
