# Supplementary figures and images for: Allogeneic cell therapy using umbilical cord MSCs on collagen scaffolds for patients with recurrent uterine adhesion: a phase I clinical trial
Source: Stem Cell Res Ther. 2018 Jul 11;9:192. doi: 10.1186/s13287-018-0904-3 (PMC6042450; doi:10.1186/s13287-018-0904-3)

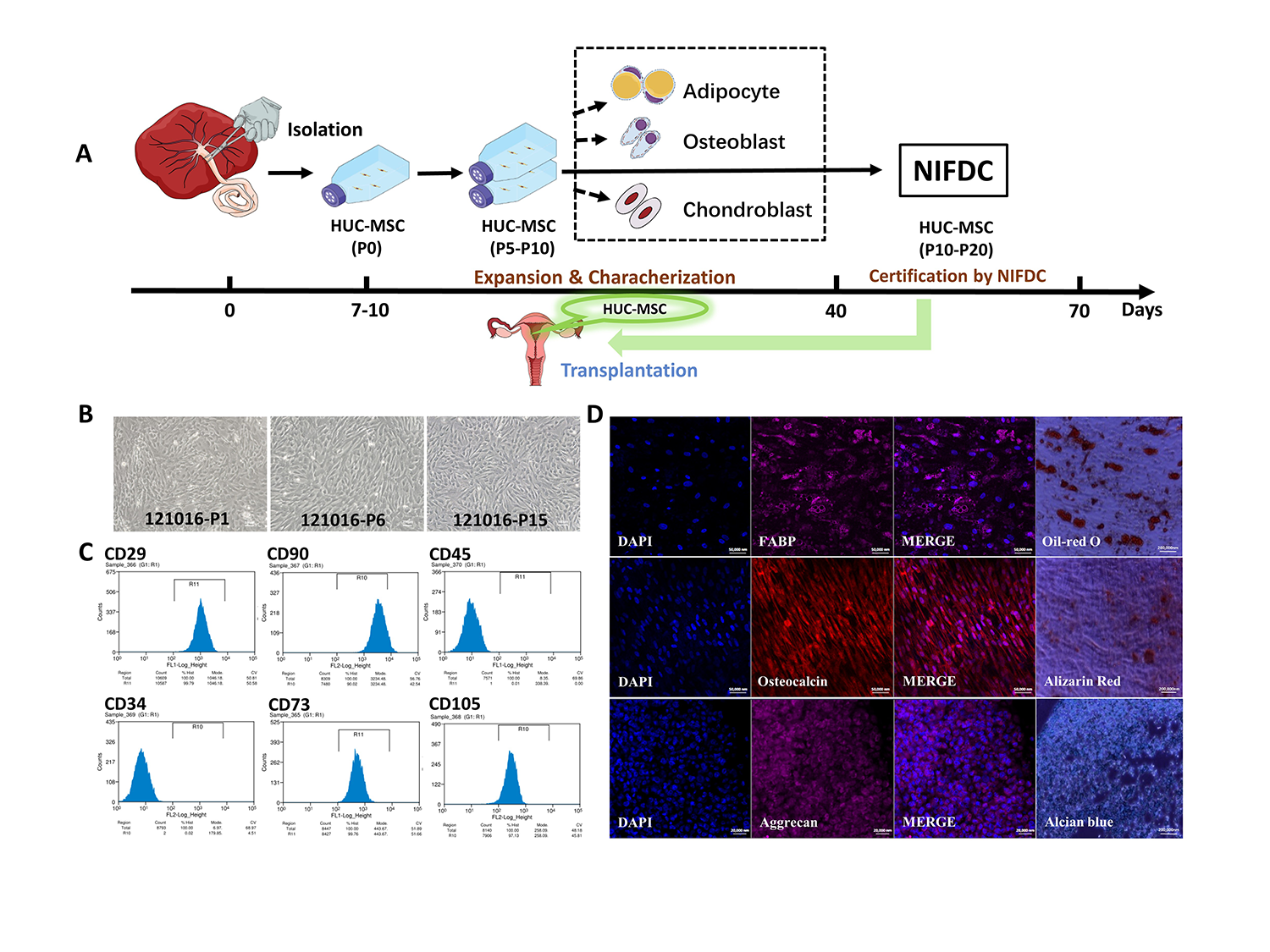

Supplement: Supplementary file 2 — Figure S1. (A) A flow chart of UC-MSC preparation for clinical use, including cell isolation, expansion, characterization, certification and safety assessment prior to transplantation. (B) Cell shapes at passages 1, 6 and 15. (C) Flow cytometry analysis of UC-MSCs showed high positive rates of CD105, CD73, CD90 and CD29, while CD45 and CD34 were negative. (D) Immunofluorescence of FABP, osteocalcin and aggrecan and immunohistochemical staining using Oil-red O, Alizarin Red and Alcian blue exhibited the presence of lipids, mineralized plaques and cartilaginous substrates derived from UC-MSCs, respectively. (TIF 4967 kb) [file 13287_2018_904_MOESM2_ESM.tif]

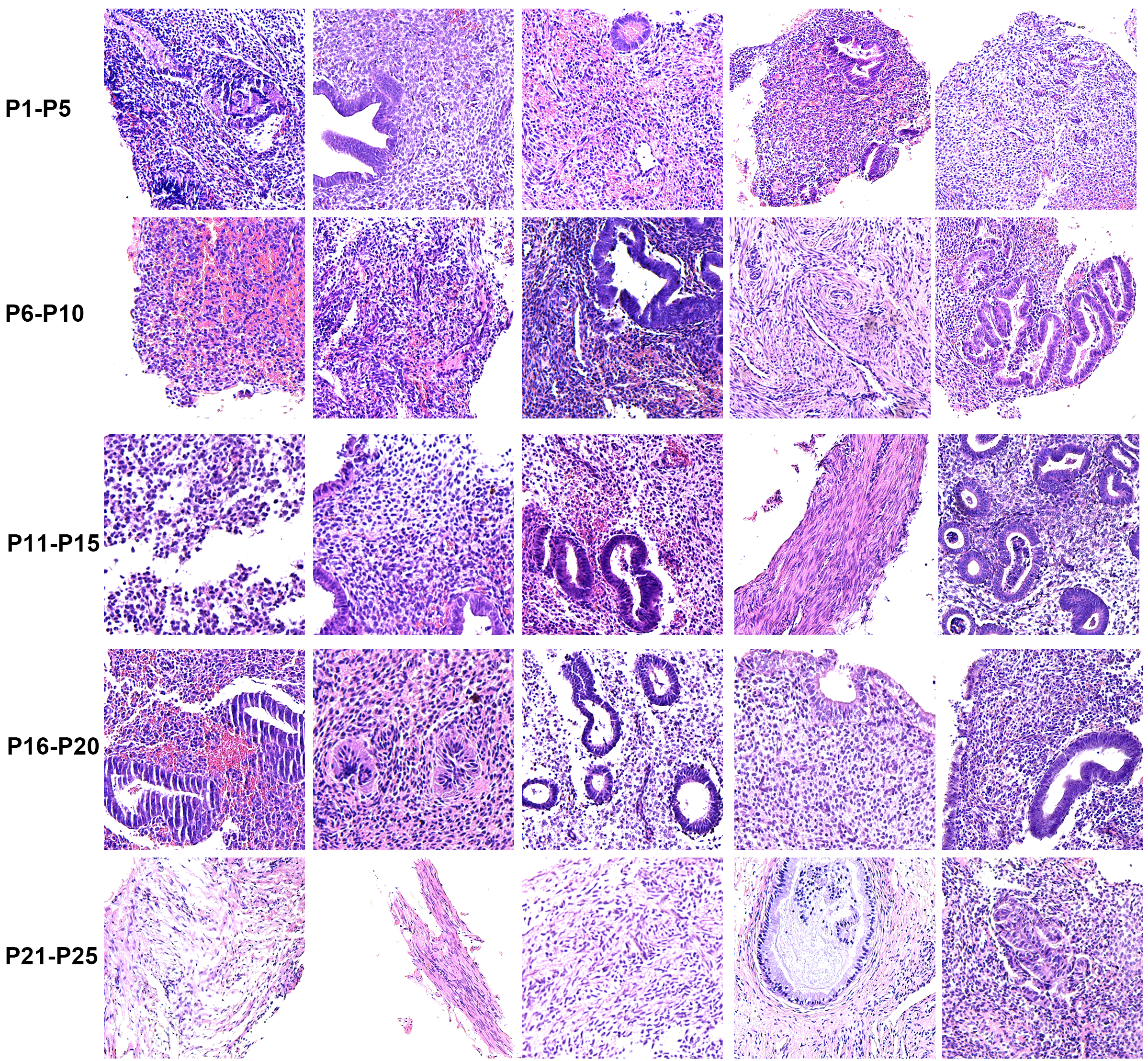

Supplement: Supplementary file 5 — Figure S4. H&E staining of endometrium biopsies from all 25 patients after UC-MSC/collagen treatment (TIF 15601 kb) [file 13287_2018_904_MOESM5_ESM.tif]

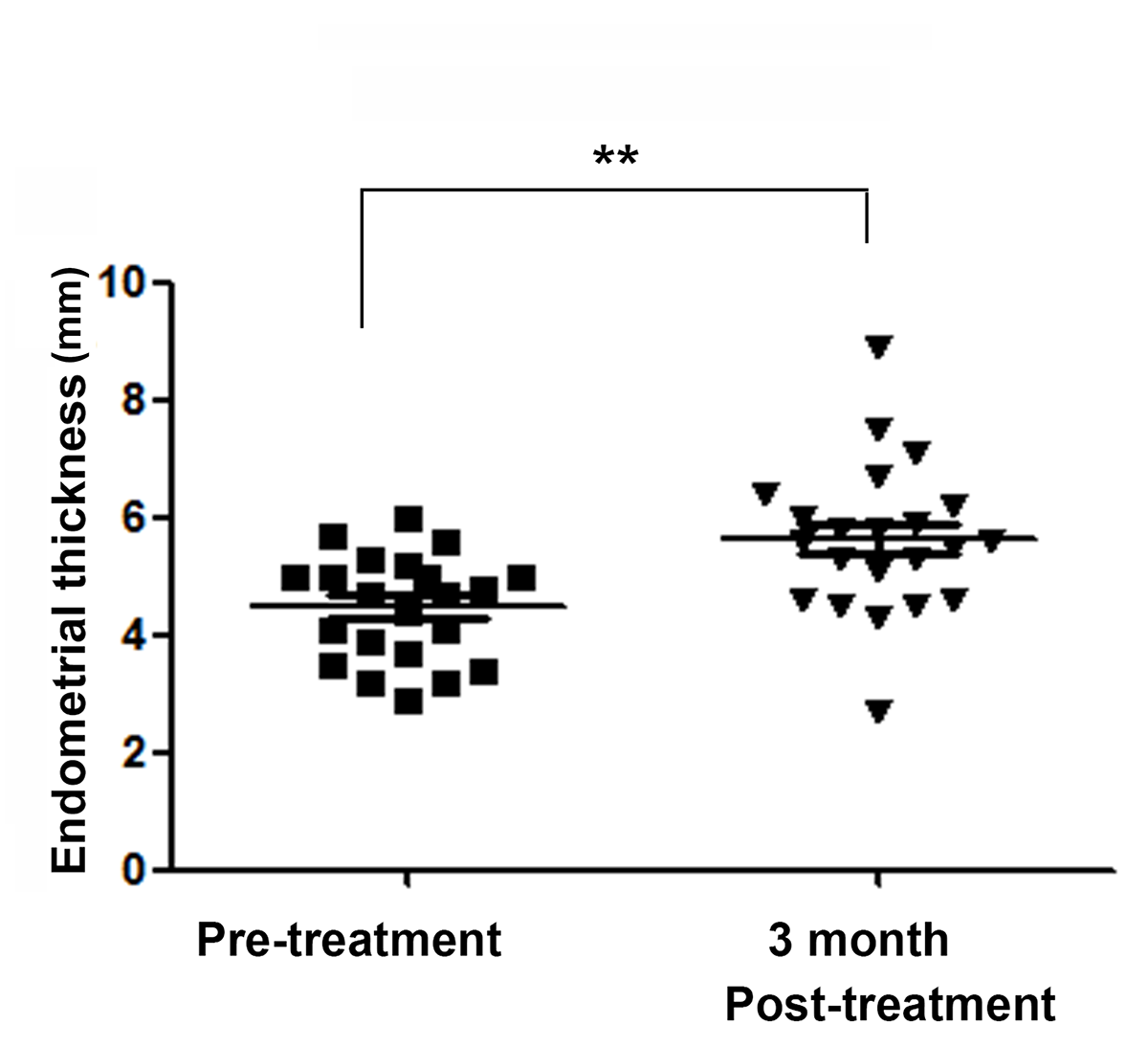

Supplement: Supplementary file 6 — Figure S2. The average maximum endometrial thickness in patients before and after UC-MSC/collagen treatment measured by ultrasound. n = 25, **P < 0.01 (TIF 3871 kb) [file 13287_2018_904_MOESM6_ESM.tif]

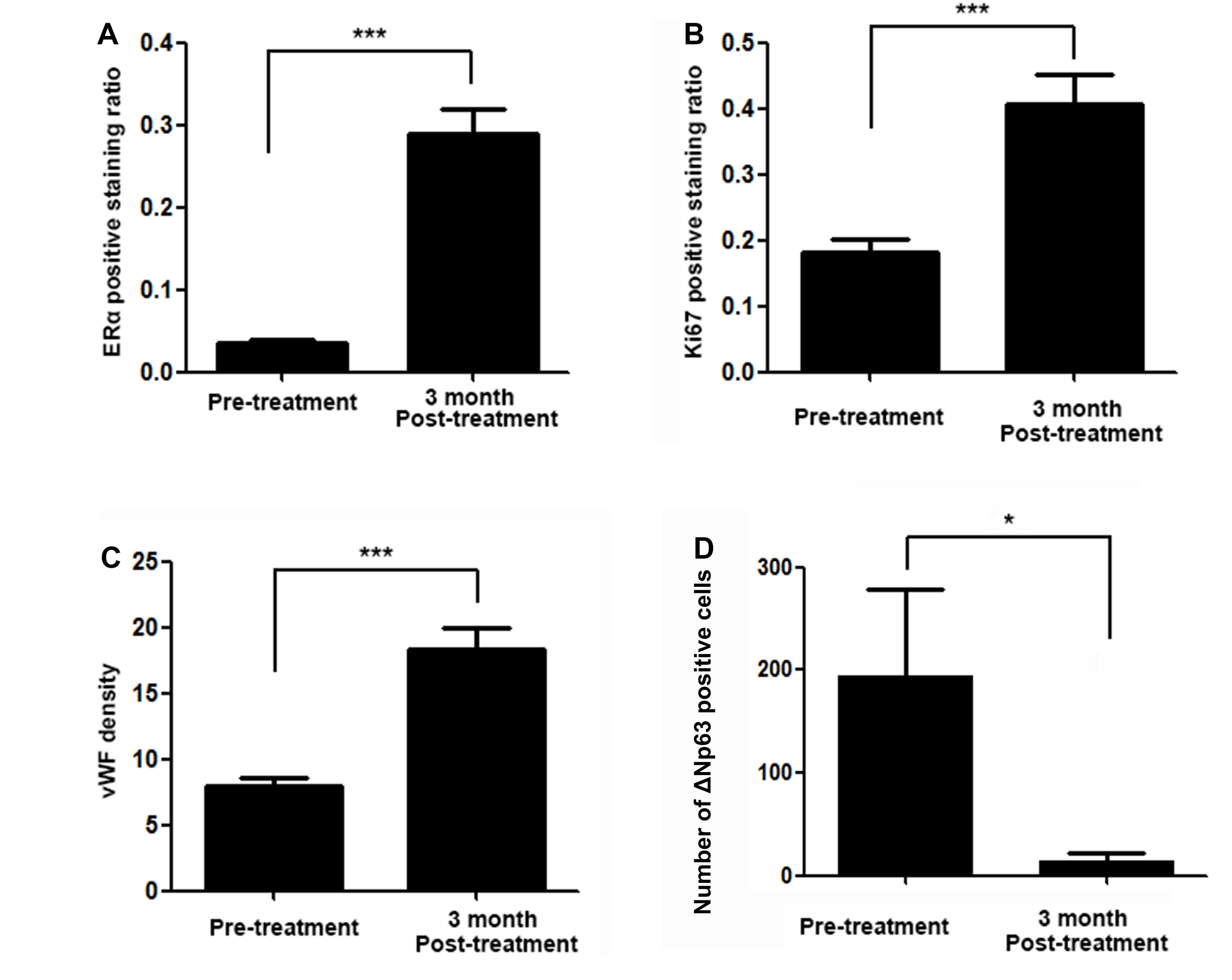

Supplement: Supplementary file 7 — Figure S3. (A-B) The positive staining ratio between ERα and Ki67 in endometrial biopsies from patients before and after UC-MSC/collagen treatment. (C) The average number of microvessels in each image of endometrial biopsies from patients before and after UC-MSC/collagen treatment. (D) The number of ΔNp63-positive cells counted in endometrial biopsies from patients before and after UC-MSC/collagen treatment. n = 25 samples, verified by two technicians, *P < 0.05, ***P < 0.001 (TIF 13448 kb) [file 13287_2018_904_MOESM7_ESM.tif]
