# Supplementary material for: Allogeneic cell therapy using umbilical cord MSCs on collagen scaffolds for patients with recurrent uterine adhesion: a phase I clinical trial
Source: Stem Cell Res Ther. 2018 Jul 11;9:192. doi: 10.1186/s13287-018-0904-3 (PMC6042450; doi:10.1186/s13287-018-0904-3)
Supplement: Supplementary file 3 — Table S1. Biological safety and biological activity analysis of the clinical-grade cells$ recognized by the National Institutes for Food and Drug Control (NIFDC). $: This table is translated from NIFDC report numbers, SH20140138 for UC-MSC. (DOCX 21 kb) [file 13287_2018_904_MOESM3_ESM.docx]

**Table S1:** Biological safety and biological activity analysis of the clinical-grade cells**^$^** recognized by the National Institutes for Food and Drug Control (NIFDC)

|  | **Contents** | **UCMSC** |
| --- | --- | --- |
| **Cell Characteristics** | **Cell morphology** | Adherent growth, fibroblast morphology |
|  | **Short tandem repeats (STRs)** | Each STR locus has 1-2 alleles |
|  | **Cell viability** | 96% |
|  | **Cell cycle analysis** | G0/G1 41.3% S 31.2% G2/M 20.9% |
|  | **Isozyme analysis** | B-type of human origin |
| **Biological safety analysis** | **Species identification and cell cross-contamination between species** | - |
|  | **Bacteria and fungi** | - |
|  | **Mycoplasma** | - |
|  | **Human papilloma virus (HPV)** | - |
|  | **Human parvovirus B19** | - |
|  | **Human immuno deficiency virus I (HIV- I)** | - |
|  | **Human immuno deficiency virus II (HIV- II)** | - |
|  | **John cunningham virus (JCV)** | - |
|  | **Epstein-barr virus (EBV)** | - |
|  | **Human hepatitis C virus (HCV)** | - |
|  | **Human hepatitis A virus (HAV)** | - |
|  | **Human cytomegalovirus (HCMV)** | - |
|  | **Human T-lymphotropic virus I (HTLV-I)** | - |
|  | **Human hepatitis B virus (HBV)** | - |
|  | **Human herpesvirus 6 (qPCR)** | - |
|  | **Human herpesvirus 7 (qPCR)** | - |
|  | **Human papillomavirus (molecular hybridization)** | - |
|  | **Reverse transcriptase activity** | - |
|  | **Bovine virus** | - |
|  | **Porcine virus** | - |
|  | **Bovine serum albumin residuals** | ＜ 5 ng/mL |
|  | **Endotoxin level** | ＜0.5 EU/mL |
|  | **Hemagglutination test of 9- to 11-day-old chick embryo allantoic fluid** | Negative |
|  | **Survival rate of 5- to 6-day-old chick embryos** | ＞90% |
|  | **Intracerebral and intraperitoneal injections in suckling mice** | Survival rate＞90% |
|  | **Intracerebral and intraperitoneal injections in mice** | Survival rate＞90% |
|  | **Intraperitoneal injection in guinea pigs** | Survival rate＞90% |
|  | **Intracutaneous and subcutaneous injections in rabbits** | Survival rate＞90% |
|  | \|  \| [**Cell surface antigen analysis**](http://www.baidu.com/link?url=_pzeZ-1a76OgdTnwavSfJ1FaypDxu2YndCV-i7odQHohYxm75IfMErZh09sEEjKnzoYtN65mJxRFo8GLQy6_Ru6yAPJVzB5lGiy3H1EH6Lyxi85wgTfyPtxDwSA7y8R-yUgIW7ePNfppA89Q5QAuza) \| \| --- \| --- \| | CD73 99.5% CD90 99.1% CD105 97.5% |
|  |  | CD11b、CD19、CD34、CD 45、HLA-DR≤2% |
| **Biological activity analysis** | **Cell differentiation ability** | Differentiated into adipocytes, osteoblasts and chondrocytes in vitro |
|  | **immunophenotypic properties** | Lymphocyte proliferation inhibition ration 50% |

***$*** This table is translated from NIFDC report numbers, SH20140138 for huc-MSC
