# Supplementary material for: Allogeneic cell therapy using umbilical cord MSCs on collagen scaffolds for patients with recurrent uterine adhesion: a phase I clinical trial
Source: Stem Cell Res Ther. 2018 Jul 11;9:192. doi: 10.1186/s13287-018-0904-3 (PMC6042450; doi:10.1186/s13287-018-0904-3)
Supplement: Supplementary file 4 — Table S2. Serum levels of C-reactive protein, leukocyte count and neutrophil percentage at 1 day after surgery in each patient. (DOCX 14 kb) [file 13287_2018_904_MOESM4_ESM.docx]

Table S2: Serum levels of C-reactive protein, leukocyte count and neutrophil percentage at 1 day after surgery in each patient

|  | leukocyte count (10^9/L) | Neutrophil percentage  (%) | C-reactive protein (mg/L) |
| --- | --- | --- | --- |
| P1 | 7.6 | 65.9 | 2.2 |
| P2 | 4.5 | 34.6 | 3.3 |
| P3 | 5.3 | 52.5 | 2.2 |
| P4 | 5.7 | 41.9 | 2.7 |
| P5 | 5.0 | 50.5 | 2.2 |
| P6 | 6.2 | 48.9 | 2.3 |
| P7 | 4.5 | 50.8 | 2.3 |
| P8 | 9.5 | 56.8 | 1.7 |
| P9 | 6.0 | 39.1 | 1.6 |
| P10 | 4.8 | 50.2 | 2.5 |
| P11 | 5.1 | 70.1 | 2.5 |
| P12 | 7.1 | 48.9 | 2.3 |
| P13 | 7.4 | 56.6 | 1.7 |
| P14 | 6.9 | 48.9 | 2.5 |
| P15 | 5.5 | 45.4 | 2.3 |
| P16 | 9.4 | 61.8 | 2.7 |
| P17 | 5.5 | 68.7 | 3.1 |
| P18 | 4.0 | 49.1 | 2.2 |
| P19 | 4.4 | 45.8 | 2.9 |
| P20 | 5.5 | 43.0 | 2.2 |
| P21 | 5.8 | 55.1 | 1.7 |
| P22 | 5.7 | 50.8 | 1.8 |
| P23 | 6.3 | 44.3 | 2.1 |
| P24 | 6.3 | 58.4 | 1.8 |
| P25 | 3.7 | 65.8 | 2.1 |
| P26 | 7.2 | 53.2 | 2.3 |
